# Albuminuria as a Risk Factor for Anemia in Chronic Kidney Disease: Result from the KoreaN Cohort Study for Outcomes in Patients With Chronic Kidney Disease (KNOW-CKD)

**DOI:** 10.1371/journal.pone.0139747

**Published:** 2015-10-02

**Authors:** Ji Suk Han, Mi Jung Lee, Kyoung Sook Park, Seung Hyeok Han, Tae-Hyun Yoo, Kook-Hwan Oh, Sue Kyung Park, Joongyub Lee, Young Youl Hyun, Wookyung Chung, Yeong Hoon Kim, Curie Ahn, Kyu Hun Choi

**Affiliations:** 1 Department of Internal Medicine, College of Medicine, Yonsei University, Seoul, Korea; 2 Department of Internal Medicine, Seoul National University, Seoul, Korea; 3 Department of Preventive Medicine, Seoul National University College of Medicine, Seoul, Korea; 4 Medical Research Collaborating Center, Seoul National University Hospital and Seoul National University College of Medicine, Seoul, Korea; 5 Department of Internal Medicine, Kangbuk Samsung Medical Center, Sungkyunkwan University, Seoul, Korea; 6 Department of Internal Medicine, Gachon University, Gil Hospital, Incheon, Korea; 7 Department of Internal Medicine, Inje University, Pusan Paik Hospital, Busan, Korea; The University of Tokyo, JAPAN

## Abstract

**Background:**

Anemia is a common complication among patients with chronic kidney disease (CKD), and it is associated with unfavorable clinical outcomes in patients with CKD independent of the estimated glomerular filtration rate (eGFR). We assessed the association of the urinary albumin-to-creatinine ratio (ACR) and eGFR with anemia in CKD patients.

**Methods:**

We conducted a cross-sectional study using baseline data from the KoreaN Cohort Study for Outcome in Patients With Chronic Kidney Disease (KNOW-CKD). Multiple regression analysis was performed to identify the independent association of albuminuria with anemia. Furthermore, odds ratios for anemia were calculated by cross-categorization of ACR and eGFR.

**Results:**

Among 1,456 patients, the mean age was 53.5 ± 12.4 years, and the mean eGFR and ACR were 51.9 ± 30.5 mL/min per 1.73 m^2^ and 853.2 ± 1,330.3 mg/g, respectively. Anemia was present in 644 patients (40.5%). Multivariate analysis showed that the odds ratio of anemia increased according to ACR levels, after adjusting for age, sex, eGFR, body mass index, pulse pressure, cause of CKD, use of erythropoiesis stimulating agents, serum calcium and ferritin (ACR < 30 mg/g as a reference group; 30–299 mg/g, adjusted odds ratio (OR) = 1.43, 95% confidence interval (CI) = 0.88–2.33; ≥300 mg/g, adjusted OR = 1.86, 95% CI = 1.12–3.10). In addition, graded associations were observed in cross-categorized groups of a higher ACR and eGFR compared to the reference group with an ACR <30 mg/g and eGFR ≥60 mL/min per 1.73 m^2^.

**Conclusion:**

The present study demonstrated that albuminuria was a significant risk factor for anemia in CKD patients independent of the eGFR.

## Introduction

The incidence and prevalence of chronic kidney disease (CKD) have rapidly increased worldwide, and CKD is recognized as a risk factor for all-cause mortality and cardiovascular mortality [1]. Anemia is a common complication in patients with CKD [2], and is associated with poorer quality of life, heart failure, and the rapid decline of renal function [3–5]. In addition, anemia is an independent risk factor for cardiovascular morbidity and mortality [4, 6]. Anemia in CKD patients is largely due to a deficiency in renal production of erythropoietin (EPO), although a deficiency of iron, folate or vitamin B12 can be another possible contributing factor. However, it remains unclear whether the main cause of anemia is a loss of EPO production capacity or a derangement in oxygen sensing [7]. Moreover, the increased prevalence of anemia in diabetic patients with albuminuria, as a marker of kidney damage, has not been explained by reduced renal function [8, 9]. When the relationship between albuminuria and anemia are elucidated, this may lead to improving the treatment of anemia.

Kidney Disease: Improving Global Outcomes (KDIGO) 2012 Clinical Practice Guideline for the Evaluation and Management of CKD recommended that CKD is classified based on the cause of CKD and the estimated glomerular filtration rate (eGFR) category, and the albuminuria category (CGA) [10]. To date, a numerous data have demonstrated that albuminuria is an independent predictor for all-cause mortality, cardiovascular disease, and CKD progression [11–14]. However, little is known about the relationship between albuminuria and anemia in CKD patients [1, 8, 15]. Therefore, in this study, we investigated whether albuminuria is significantly associated with anemia in patients with CKD.

## Materials and Methods

### Ethics statement

The study protocol was approved by the institutional review board at each participating clinical center including the Seoul National University Hospital, Severance Hospital, Kangbuk Samsung Medical Center, Seoul St. Mary’s Hospital, Gil Hospital, Eulji General Hospital, Chonnam National University Hospital and Pusan Paik Hospital in 2011. All participating patients provided written informed consent.

### Subjects

We used baseline data of the KoreaN Cohort Study for Outcome in Patients With Chronic Kidney Disease (KNOW-CKD), a nationwide prospective cohort including CKD stage 1–5 non-dialysis patients from February 2011 to July 2014. The study aimed to explore the risk factors for renal or cardiovascular outcomes in Korean CKD populations. The detailed design and methods of the KNOW-CKD have been previously published [16]. Among 1,528 patients initially included, 72 were excluded due to following reasons: a missing eGFR or urinary albumin-to-creatinine ratio (ACR) data in 58 patients and missing hemoglobin levels in 14. Finally, 1,456 patients were analyzed in the final analysis.

### Data collection

Baseline demographics, and clinical and laboratory values were extracted from the electronic data management system (PhactaX). Blood samples were analyzed at each hospital laboratory of the participating centers. Ten mL of whole blood was obtained by using the serum separation tube (SST) and centrifuged within 1 hour for serum separation before being sent to the central laboratory (Lab Genomics) for creatinine measurement. Serum creatinine was measured by an IDMS-traceable method and the eGFR was estimated using the CKD Epidemiology Collaboration equation using age, sex, race, and the serum creatinine level [17]. Another 15 mL of the first-voided urine was also collected to analyze urinary concentrations of albumin and creatinine at the central laboratory, and the ACR was expressed as mg/g. Albuminuria was determined by urinary ACR from spot urine samples. Subjects were categorized into three groups according to the ACR (A1, A2, and A3), which were defined as ACR <30, 30–299, and ≥300 mg/g, respectively. In addition, we measured the protein excretion rate using timed urine collection at each respective hospital. Anemia was defined using the World Health Organization guideline: hemoglobin <12 g/dL for women and <13 g/dL for men [18]. The serum iron, total iron-binding capacity (TIBC), and serum ferritin were measured, and the transferrin saturation (TSAT, %) was calculated as (iron/TIBC) × 100. The body mass index (BMI) was calculated as weight in kg divided by the square of height in m. Two-dimensional echocardiography was performed to measure cardiac parameters, and the left ventricular mass index (LVMI) was calculated by dividing the LV mass by the body surface area at each respective hospital.

### Statistical analyses

Continuous variables were expressed as mean ± standard deviation and categorical variables as a number (percentage). The ACR was analyzed as a categorical variable and as a natural log-transformed variable (Ln ACR) to approximate a normal distribution. To compare baseline characteristics according to the ACR groups, analysis of variance and chi-square tests were used for continuous variables and categorical variables, respectively. Spearman’s correlation analysis was used to determine the association between hemoglobin and Ln ACR. We performed univariate logistic regression analysis to determine the association between anemia and other variables including demographics and various laboratory data. Multivariate logistic regression analysis was applied to calculate adjusted odds ratio (OR) by stepwise backward selection with adjustment for covariate effects to those variables that were significantly associated with anemia in univariate analysis. Missing variables were replaced using the linear interpolation imputation method. Moreover, ORs were ascertained by model approaches for covariate adjustment as follows: model 1 = adjusted for age and sex; model 2 = model 1 + eGFR; and mode 3 = model 2 + cause of CKD, BMI, use of an erythropoiesis stimulating agent (ESA), pulse pressure, serum calcium, and ferritin. Graphical illustrations of the relationship of the ACR and eGFR with anemia were also obtained by generalized additive models. Additionally, we evaluated multiplicative interaction based on categories of the combination of eGFR and ACR. We compared the risk in nine categories of the eGFR (<45, 45–59, ≥60 mL/min per 1.73 m^2^) and albuminuria (ACR <30, 30–299, ≥300 mg/g). Generalized additive models were computed using the R Statistical package software (version 2.15.3), and SPSS software, version 19.0 (SPSS Inc.) was used to perform the remaining statistical analyses. Tests were considered statistically significant at P < 0.05.

## Results

### Baseline characteristics of subjects

Among 1,456 patients, the mean age was 53.5 ± 12.4 years, and 896 patients (61.5%) were male. The mean eGFR and ACR were 51.9 ± 30.5 mL/min per 1.73 m^2^ and 853.2 ± 1,330.3 mg/g, respectively. Glomerulonephritis (35.9%) was the most common cause of CKD, followed by diabetic nephropathy (21.6%), hypertensive nephropathy (20.2%), and polycystic kidney disease (17.4%). Two hundred twenty two patients (15.2%) had an eGFR ≥90 mL/min per 1.73 m^2^, 268 (18.4%) had an eGFR of 60–89 mL/min per 1.73 m^2^, 227 (15.6%) had an eGFR of 45–59 mL/min per 1.73 m^2^, 316 (21.7%) had an eGFR of 30–44 mL/min per 1.73 m^2^, 317 (21.8%) had an eGFR of 15–29 mL/min per 1.73 m^2^, and 106 (7.3%) had an eGFR <15 mL/min per 1.73 m^2^. A2 (ACR 30–299 mg/g) and A3 (ACR ≥300 mg/g) were present in 455 (31.3%) and 774 (53.2%) patients, respectively. The mean hemoglobin levels were 12.8 ± 1.9 g/dL, and 644 patients (44.2%) had anemia at baseline. One hundred-twenty seven patients (8.7%) were taking ESA therapy. Baseline clinical characteristics stratified by categories of ACR are presented in Table 1. Subjects with macroalbuminuria were more likely to have diabetes mellitus and anemia. The systolic blood pressure, LVMI, BMI, and parathyroid hormone were significantly higher in the higher–ACR group. Meanwhile, the eGFR and levels of serum albumin, calcium, and total cholesterol were significantly lower in the higher ACR group. No significant difference in age, sex, smoking history, and C-reactive protein, and serum ferritin levels was detected among the three groups. Baseline demographics and the laboratory value according to the cause of CKD are shown in S1 Table.

**Table 1:** Baseline characteristics of subjects according to albuminuria.

Characteristics	ACR (mg/g)			*P*-value
	<30 (N = 227)	30–299 (N = 455)	≥300 (N = 774)	
	Mean (SD)	Mean (SD)	Mean (SD)	
Age (years)	52.8 ± 13.4	53.5 ± 12.1	53.7 ± 12.2	0.59
BMI (kg/m^2^)	23.9 ± 3.2	24.1 ± 3.2	24.8 ± 3.4	<0.001
SBP (mmHg)	125.1 ± 14.2	125.7 ± 15.0	130.1 ± 17.3	<0.001
DBP (mmHg)	76.8 ± 10.9	76.0 ± 10.4	77.2 ± 11.5	0.18
MAP (mmHg)	92.9 ± 11.1	92.6 ± 10.9	94.9 ± 12.3	<0.01
Pulse pressure (mmHg)	48.3 ± 10.0	49.6 ± 11.4	52.9 ± 12.7	<0.001
eGFR (mL/min per 1.73 m^2^)	72.8 ± 30.1	53.2 ± 28.6	44.9 ± 28.7	<0.001
PCR (mg/g)	53.0 ± 43.4	216.6 ± 133.6	2,205.7 ± 2,398.3	<0.001
24-h urine protein (mg/day)	143.6 ± 640.4	314.7 ± 299.1	2,132.4 ± 2,178.0	<0.001
Hemoglobin (g/dL)	13.77 ± 1.56	12.96 ± 1.84	12.46 ± 2.07	<0.001
Ferritin (ng/mL)	143.3 ± 133.9	136.9 ± 143.1	138.0 ± 148.5	0.86
TSAT (%)	33.3 ± 13.1	30.9 ± 11.7	31.3 ± 12.3	0.05
Albumin (g/dL)	4.40 ± 0.26	4.31 ± 0.29	4.04 ± 0.44	<0.001
Total cholesterol (mg/dL)	174.6 ± 32.3	169.2 ± 33.2	175.7 ± 40.9	0.01
Calcium (mg/dL)	9.27 ± 0.37	9.21 ± 0.46	9.01 ± 0.58	<0.001
Phosphorus (mg/dL)	3.50 ± 0.52	3.57 ± 0.59	3.84 ± 0.71	<0.001
PTH (pg/mL)	50.9 ± 48.4	64.3 ± 54.8	87.4 ± 90.3	<0.001
Uric acid (mg/dL)	6.13 ± 1.90	6.99 ± 1.91	7.54 ± 1.91	<0.001
CRP (mg/L)	0.15 ± 0.37	0.22 ± 0.59	0.20 ± 0.51	0.26
LVMI (g/m^2^)	87.3 ± 22.1	91.9 ± 25.1	99.0 ± 30.3	<0.001
	N (%)	N (%)	N (%)	
Male sex	148 (65.2%)	268 (58.9%)	480 (62.0%)	0.26
Smokers	45 (19.8%)	68 (15.0%)	127 (16.4%)	0.28
Cause of CKD				<0.001
DM	9 (4.0%)	67 (14.7%)	240 (31.0%)	
HTN	72 (31.7%)	96 (21.1%)	129 (16.7%)	
GN	24 (10.6%)	148 (32.5%)	345 (44.6%)	
PKD	108 (47.6%)	125 (27.5%)	22 (2.8%)	
Unclassified	14 (6.2%)	19 (4.2%)	38 (4.9%)	
Hypertension	196 (89.1%)	414 (94.3%)	718 (95.7%)	0.001
Diabetes mellitus	30 (13.6%)	108 (24.6%)	329 (43.9%)	<0.001
Cardiovascular disease	29 (13.2%)	72 (16.4%)	126 (16.8%)	0.43
Use of iron agent	16 (7.1%)	66 (14.6%)	132 (17.1%)	0.001
Use of ESA	7 (3.1%)	29 (6.4%)	91 (11.8%)	<0.001
ACEI use	18 (9.0%)	43 (10.0%)	117 (15.4%)	<0.01
ARB use	157 (78.9%)	360 (83.5%)	628 (82.8%)	0.34

Data are expressed as mean (with standard deviation) or number (%).

*Abbreviations*: ACR, albumin-to-creatinine ratio; ACEI, angiotensin converting enzyme inhibitor; ARB, angiotensin receptor blocker; BMI, body mass index; CKD, chronic kidney disease; CRP, C-reactive protein; CVD, cardiovascular disease; DBP, diastolic blood pressure; DM, diabetic mellitus; eGFR, estimated glomerular filtration rate; ESA, erythropoiesis stimulating agent; GN, glomerulonephritis; HTN, hypertension; LVMI, left ventricular mass index; MAP, mean arterial pressure; PCR, protein-to-creatinine ratio; PKD, polycystic kidney disease; PTH, parathyroid hormone; SBP, systolic blood pressure; TSAT, transferrin saturation.

### Associations between anemia and other variables

**Fig 1:**
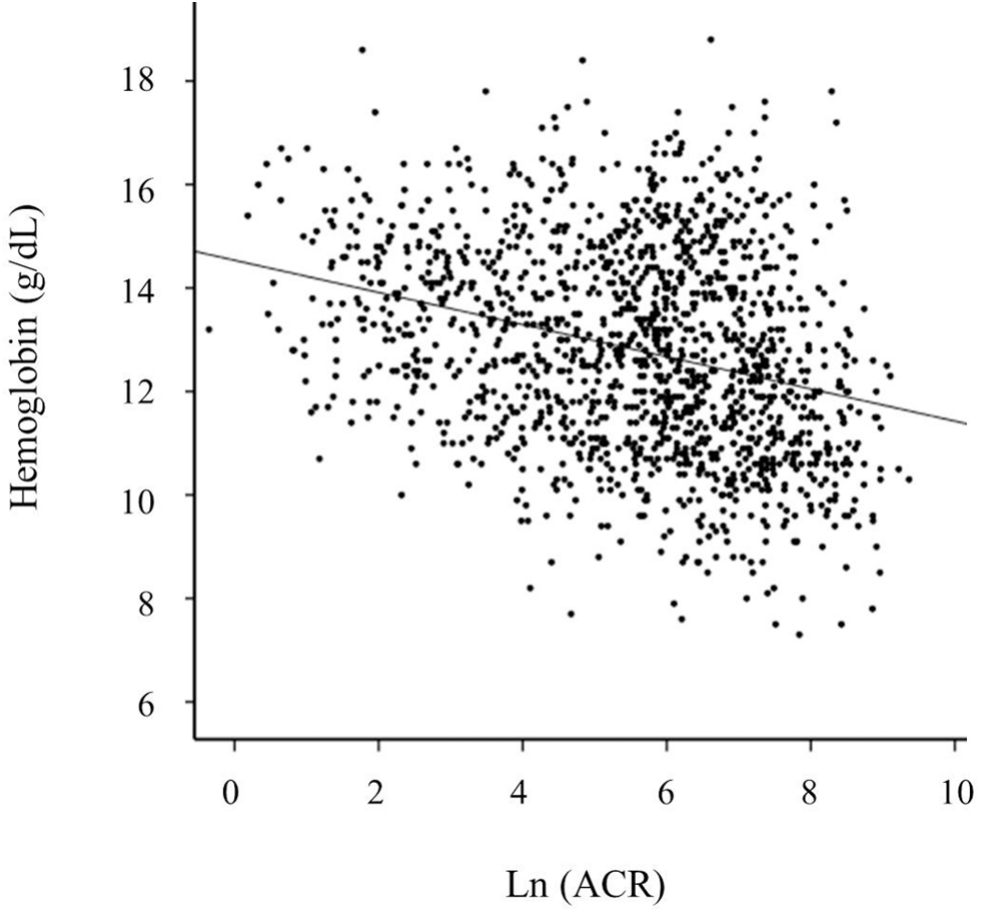
Correlation of hemoglobin levels with albuminuria. Hemoglobin levels are inversely related with Ln ACR levels (r = -0.281, P < 0.001). *Abbreviation*: ACR, albumin-to-creatinine ratio; Ln ACR, log-transformed albumin-to-creatinine ratio

Pearson’s correlation analysis showed that hemoglobin levels were inversely related with Ln ACR levels (r = -0.296, P < 0.001) (Fig 1). Univariate logistic regression was performed to analyze associations between anemia and other variables (S1 Table). We found that old age, female sex, diabetes mellitus, a previous history of cardiovascular disease, and non-current smokers were significantly associated with higher odds of anemia. CKD patients with the lowest quintile of TSAT and the lowest or the highest quintile of ferritin were significantly associated with anemia. A higher pulse pressure, LVMI, and serum uric acid and phosphorus levels were associated with anemia, whereas a lower BMI and lower levels of serum albumin, cholesterol, and calcium were related to anemia.

### Albuminuria as an independent risk factor for anemia

The prevalence rates and multivariable-adjusted ORs for anemia according to the ACR categories are presented in Table 2. Crude prevalence rates of anemia according to the ACR increased across a higher ACR group. When adjusted for the eGFR, age and sex, populations with macroalbuminuria had a two fold increase in the OR of anemia compared to those with normoalbuminuria. Furthermore, in the fully adjusted model, macroalbuminuria was still associated with an increased risk of anemia. This analysis was considerably powered enough (adjusted R^2^ for fully adjusted model was 0.387). Otherwise, microalbuminuria failed to show a significant increase in the prevalence of anemia compared with the reference group. When patients using an ESA were regarded as those with anemia or were excluded in multivariate analysis, the value of OR for anemia was not noticeably changed (S3 and S4 Tables).

**Table 2:** Odds ratios for anemia associated with ACR.

	Prevalence rates	OR (95% CI)		
ACR (mg/g)	N (%)	Model 1	Model 2	Model 3
<30	44 (19.4%)	1 (reference)	1 (reference)	1 (reference)
30–299	181 (39.8%)	2.69 (1.83–3.96)	1.48 (0.96–2.28)	1.43 (0.88–2.33)
**≥**300	419 (54.1%)	5.00 (3.47–7.21)	2.11 (1.40–3.17)	1.86 (1.12–3.10)

Anemia (hemoglobin <13 g/dL for men, <12 g/dL for women).

Model 1: adjusted for age and sex (adjusted R^2^ = 0.106, *P* = 0.028)

Model 2: adjusted for age, sex, and the eGFR (adjusted R^2^ = 0.296, *P* = 0.123)

Model 3: adjusted for age, sex, the eGFR, serum calcium level, BMI, pulse pressure, use of ESA, smoking, the cause of CKD, and ferritin level (adjusted R^2^ = 0.387, *P* = 0.005)

*Abbreviations*: ACR, albumin creatinine ratio; eGFR, estimated GFR; CI, confidence interval; BMI, body mass index; ESA, erythropoiesis stimulating agent; CKD, chronic kidney disease.

**Fig 2:**
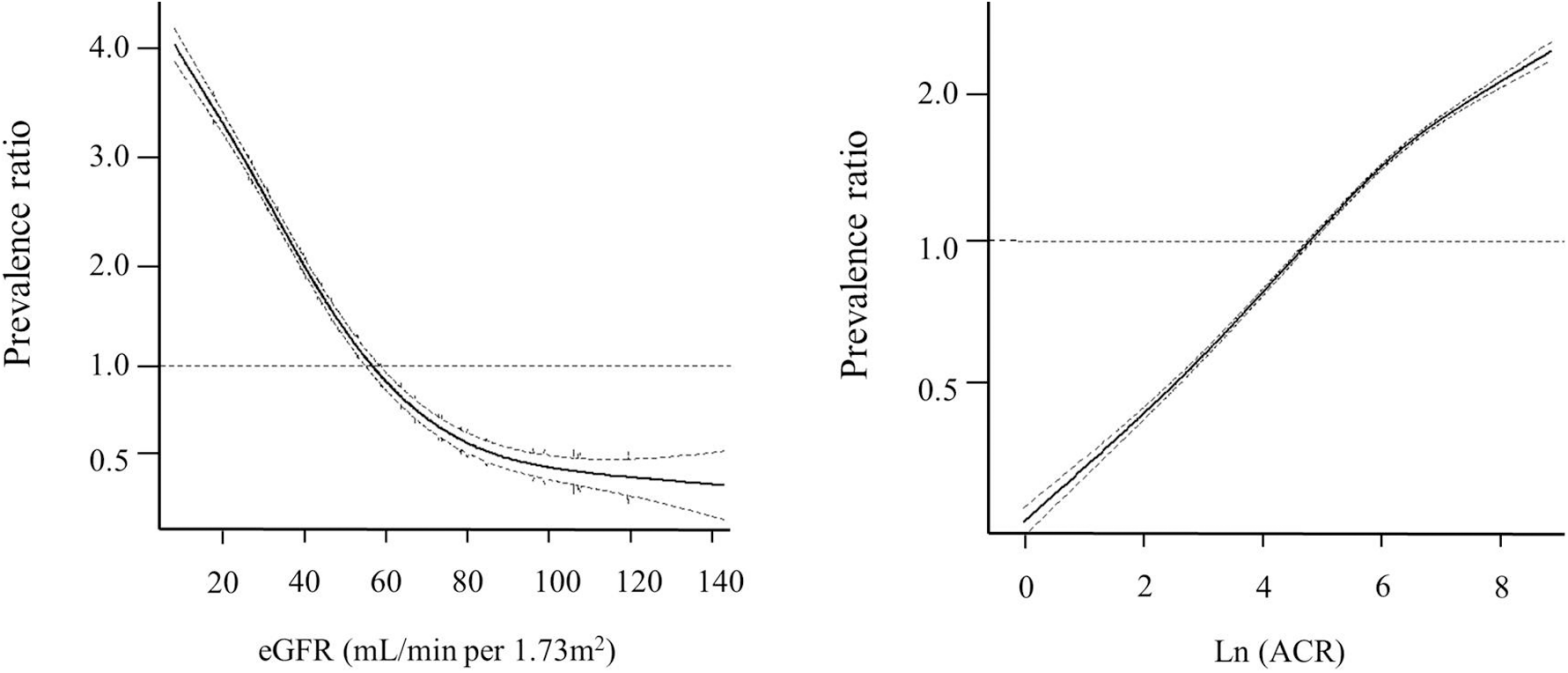
The risk of anemia in relation to the ACR and eGFR. By using generalized additive models, the risk (solid line) and 95% confidence intervals (dashed line) of anemia in relation to the (A) ACR and (B) eGFR is estimated. *Abbreviations*: ACR, albumin-to-creatinine ratio; eGFR, estimated glomerular filtration rate.

The adjusted ORs for anemia associated with ACR, as a continuous variable, were calculated using generalized additive models. Fig 2 shows that lower levels of the eGFR and higher levels of the ACR were associated with anemia after adjusting for age, sex, the ACR (for the eGFR), and the eGFR (for the ACR), as continuous variables.

In multivariate regression analysis, variables that were significantly related to anemia are presented in S5 Table.

### Joint associations of albuminuria and the eGFR with anemia

We further examined the joint association between albuminuria and the eGFR with the risk of anemia by calculating ORs based on the cross-categorization of ACR and eGFR (Table 3). Compared to the reference groups of eGFR ≥60 mL/min per 1.73 m^2^ and ACR <30 mg/g, the ORs for anemia of groups with an ACR 30–299 mg/g or ACR ≥300 mg/g in each eGFR category showed an increasing trend according to the ACR.

**Table 3:** Odds ratios for anemia based on the cross-categorization of albuminuria and the eGFR.

eGFR (mL/min per 1.73 m^2^)	ACR (mg/g)	Non-anemia (N = 812)	Anemia (N = 644)	OR (95% CI)^1^
≥60	<30	126 (15.5)	12 (1.9)	1 (reference)
	30–299	131 (16.1)	23 (3.6)	1.88 (0.86–4.09)
	≥300	159 (19.6)	39 (6.1)	3.10 (1.42–6.77)
45–59	<30	29 (3.6)	10 (1.5)	4.51 (1.62–12.57)
	30–299	56 (6.9)	26 (4.0)	5.30 (2.34–12.01)
	≥300	63 (7.8)	43 (6.7)	7.39 (3.30–16.55)
<45	<30	28 (3.4)	22 (3.4)	7.75 (3.09–19.42)
	30–299	87 (10.7)	132 (20.5)	13.30 (6.50–27.25)
	≥300	133 (16.4)	337 (52.3)	18.73 (9.06–38.75)

^1^ Adjusted for age, sex, the cause of CKD, BMI, pulse pressure, ferritin and serum calcium levels, and the use of an ESA (adjusted R^2^ = 0.357, *P* = 0.187)

*Abbreviations*: ACR, albumin-to-creatinine ratio; BMI, body mass index; CI, confidence interval; CKD, chronic kidney disease; eGFR, estimated glomerular filtration rate; ESA, erythropoiesis stimulating agent; OR, odds ratio.

## Discussion

The results of this study showed that albuminuria was significantly associated with a higher prevalence of anemia in patients with CKD, independent of the eGFR. Furthermore, graded associations were also found in the cross-categorized group of macroalbuminuria (ACR ≥ 300 mg/g) compared with normoalbuminuria (ACR < 30 mg/g) in every eGFR category.

Anemia is a common manifestation of CKD and the prevalence of anemia increases as the eGFR declines [2]. Anemia is associated with a poorer quality of life and the rapid decline of renal function, as well as cardiovascular mortality [3, 4, 19]. Anemia is mainly caused by insufficient kidney EPO production and a deficiency of the available iron to support ongoing erythropoiesis in CKD patients. However, several different factors including inflammation, malnutrition, and metabolic disease, have been considered to contribute to the anemia of CKD. Besides, previous studies have demonstrated that people with diabetes were more likely to have anemia compared to those without diabetes [8, 9]. Furthermore, anemia was more frequently present in a diabetic population with albuminuria compared to those without albuminuria [8, 9]. To date, several studies have demonstrated that albuminuria is a strong and independent predictor for the progression of kidney disease, development of cardiovascular disease, and mortality [11, 14, 20, 21]. The predictive ability of albuminuria at all categories of the eGFR suggested that albuminuria categories should be added to the CKD staging system [10]. However, a limited number of data were available for the association between albuminuria and anemia [1, 8, 15]. In the National Health and Nutrition Examination Survey (NHANES) data from the United States, anemia was associated with the ACR at certain eGFR strata (40–59 and ≥120 mL/min per 1.73 m^2^) in a general population of 30,528 people[1]. However, although this study had a large population size, there were few people with decreased renal function (5.5%, eGFR <60 mL/min per 1.73 m^2^) and macroalbuminuria (3.1%, ACR ≥300 mg/g). In another cross-sectional study that included 820 patients with diabetes, the prevalence of anemia were stratified according to the albumin excretion rate (<20, 20–200, and >200 μg/min) and eGFR (<60, 60–80, and >80 mL/min per 1.73 m^2^). Subjects with macroalbuminuria were more likely to have anemia at all the eGFR categories [8]. Furthermore, in the Chronic Renal Insufficiency Cohort (CRIC) study, a multicenter observational study of 3,030 participants with mild to severe CKD, a higher ACR and urinary protein creatinine ratio were associated with lower serum hemoglobin levels. However, subjects with preserved eGFR were not included in the CRIC study [15]. Unlike the NHANES data, the present study found that there was a graded increase between anemia and albuminuria when the ACR and eGFR were cross-categorized. However, the CRIC study showed an association between ACR and level of serum hemoglobin after adjusting for the eGFR; in our study, anemia was defined by the World Health Organization, and it was related to albuminuria when further adjusted for age, sex, eGFR, and iron status.

The increased prevalence of anemia in diabetic patients with albuminuria cannot be explained by reduced renal function [8, 9]. Some authors have proposed that significant losses of transferrin in heavy proteinuria resulted in iron-deficiency anemia [22, 23]. Others have suggested that endothelial dysfunction and microvascular damage in the renal tubulointerstitium which were thought to be the pathogenesis of albuminuria, can lead to the impairment of EPO production and release [24–27]. Tubulointerstitial injury is a major feature of diabetic nephropathy and also reflects influences in other forms of renal disease[28]. Unal et al. found that anemia was frequent among kidney transplant recipients with microalbuminuria and they suggested that a tubulointerstitial injury of chronic allograft nephropathy leads to erythropoietin deficiency, which starts even before significant deterioration of excretory renal function has occurred [29]. In addition, a recent study demonstrated that a low hemoglobin level predicted the progression or development of albuminuria in those with type 2 diabetes [30]. The authors proposed that the leakage of protein which is thought to be an important contributor to progressive tubulointerstitial injury, may cause ineffective erythropoiesis in renal tubulointerstitium. Indeed, further experimental studies are needed to explore the delicate interaction mechanisms between impaired endothelial function and erythropoiesis. Based on these findings, we surmised that anemia, an upcoming early marker of microvascular damage and tubulointerstitial injury, can precede declining renal function, similar to albuminuria. However, this issue should be confirmed by further studies on the serial correlation between changes in renal function and hemoglobin levels. In the present study, according to the recommendations of KDIGO 2012 Clinical Practice Guideline for the Evaluation and Management of CKD, we classified participants according to the cause of CKD, eGFR, and albuminuria categories, and found that there was a graded association between albuminuria and anemia. In addition, diabetes mellitus, as a cause of CKD, was independently associated with higher odds of anemia irrespective of the eGFR and albuminuria. Therefore, our finding can serve as additional evidence to support the CGA staging system (the cause, GFR, and albuminuria) by KDIGO.

In our study, serum ferritin and TSAT levels, as measures of the iron deficiency status, were feasible in most of the study subjects. The lowest TSAT quintile, and the lowest and highest serum ferritin quintiles showed a high OR for anemia. Measuring TSAT and the serum ferritin are well-established tests for assessing iron status. However, since serum ferritin is an acute-phase reactant, high ferritin concentrations should be interpreted with caution. In the present study, there was a correlation between serum ferritin levels and the Ln C-reactive protein level (r = 0.104, P < 0.001). A U-shaped relationship between the serum ferritin level and anemia was also observed. Although serum ferritin levels were included in multivariable analysis, the association between anemia and albuminuria remained significant. Compared to previous studies, reliable data and adjusting the iron deficiency status could be the strengths of this study. Meanwhile, considering the association between anemia and albuminuria, it can be hypothesized that recognizing of patients with albuminuria and the use of interventions such as angiotensin converting enzyme inhibitors (ACEI) or an angiotensin receptor blocker (ARB) may lead to the improvement of anemia. However, ACEI has been suggested as causing anemia in several studies rather than improving anemia [31, 32]. In a retrospective analysis from electronic health record data of 701 patients receiving ACEI or an ARB, the use of ACEI was associated with decreased hemoglobin levels compared with an ARB [31]. In another retrospective cohort study including 14,754 patients taking ACEI and 751 patients taking ARB, hemoglobin levels were reduced during the first year of using ACEI and and ARB [32]. In the current study, ACEI (versus non-user, OR = 1.06, 95% CI = 0.77–1.45, P = 0.73) and ARB (versus non-user, OR = 1.10, 95% CI = 0.83–1.45, P = 0.52) were not significantly associated with anemia. Based on these findings, we inferred that there were no definitive conclusions of the role of ACEI or an ARB on anemia. The therapeutic use of ACEI or an ARB for anemia may need to be investigated in a further prospective randomized controlled study.

There are several limitations of this study. First, only a single ACR measurement was available at baseline. ACR level may be high due to extremely low levels of creatinine as well as high levels of albuminuria. However, the 24-hour urine collection was obtained from most of the study population, and the protein excretion rate was measured, showing excellent correlation with the ACR. Although timed urine collection (usually for 24 h) remains the gold standard assessment of proteinuria, measurement of the ACR is more convenient, and it showed superiority in predicting renal events [33]. Second, more than a third of the study population had CKD caused by glomerulonephritis. This is because glomerulonephritis was defined by the presence of glomerular hematuria or albuminuria with or without underlying systemic disease causing glomerulonephritis. Third, this is a cross-sectional analysis, so we were not able to determine causality in the relationship between albuminuria and anemia. Finally, since the prevalence of anemia is very low in populations with an eGFR >90 mL/min per 1.73 m^2^, patients with eGFR ≥60 mL/min per 1.73 m^2^ were not further categorized into >90 and 60–89 mL/min per 1.73 m^2^ group according to CKD staging recommendations in multiplicative categorization between the eGFR and albuminuria [10, 21]. Likewise, since the number of subjects with a decreased eGFR (<30 ml/min per 1.73m^2^) and normoalbuminuria was small, groups with eGFR <45 mL/min per 1.73 m^2^ were not further categorized into CKD stages 3b, 4, and 5. Notwithstanding these limitations, the simultaneous measurement of the ACR and creatinine in a central laboratory and the enrollment of CKD patients with a preserved eGFR are the major strengths of this study.

In conclusion, the present study demonstrated that albuminuria was significantly associated with an increased prevalence of anemia, independently of eGFR, in patients with CKD.

## Supporting Information

S1 Table Univariate logistic regression analysis for the risk of anemia. *Abbreviations*: ACR, albumin to creatinine ratio; ACEI, angiotensin converting enzyme inhibitor; ARB, angiotensin receptor blocker; BMI, body mass index; CI, confidence interval; CKD, chronic kidney disease; DN, diabetic nephropathy; eGFR, estimated glomerular filtration rate; ESA, erythropoiesis-stimulating agent; GN, glomerulonephritis; HTN, hypertension; LVMI, left ventricular mass index; OR, Odds ratio; PCR, protein to creatinine ratio; PKD, polycystic kidney disease; PTH, parathyroid hormone; SBP, systolic blood pressure; TSAT, transferrin saturation.
(DOCX)

S2 Table Univariate logistic regression analysis for the risk of anemia. *Abbreviations*: ACR, albumin to creatinine ratio; ACEI, angiotensin converting enzyme inhibitor; ARB, angiotensin receptor blocker; BMI, body mass index; CI, confidence interval; CKD, chronic kidney disease; DN, diabetic nephropathy; eGFR, estimated glomerular filtration rate; ESA, erythropoiesis-stimulating agent; GN, glomerulonephritis; HTN, hypertension; LVMI, left ventricular mass index; OR, Odds ratio; PCR, protein to creatinine ratio; PKD, polycystic kidney disease; PTH, parathyroid hormone; SBP, systolic blood pressure; TSAT, transferrin saturation.
(DOCX)

S3 Table Odds ratio for anemia associated with ACR. Anemia (hemoglobin <13 g/dL for men, <12 g/dL for women or with use of ESA). Model 1: adjusted for age and sex. Model 2: adjusted for age, sex, and the eGFR. Model 3: adjusted for age, sex, the eGFR, serum calcium level, BMI, use of an ESA, smoking, the cause of CKD, and ferritin level. *Abbreviations*: ACR, albumin creatinine ratio; eGFR, estimated GFR; CI, confidence interval; BMI, body mass index; ESA, erythropoiesis stimulating agent; CKD, chronic kidney disease.
(DOCX)

S4 Table Odds ratio for anemia associated with ACR (patients with ESA are excluded). Anemia (hemoglobin <13 g/dL for men, <12 g/dL for women). Model 1: adjusted for age and sex**.** Model 2: adjusted for age, sex, and the eGFR**.**Model 3: adjusted for age, sex, the eGFR, serum calcium level, BMI, use of an ESA, smoking, the cause of CKD, and ferritin level**.** *Abbreviations*: ACR, albumin creatinine ratio; eGFR, estimated GFR; CI, confidence interval; BMI, body mass index; ESA, erythropoiesis stimulating agent; CKD, chronic kidney disease.
(DOCX)

S5 Table Multivariate logistic regression analysis for the risk of anemia. *Abbreviations*: ACR, albumin to creatinine ratio; BMI, body mass index; CI, confidence interval; CKD, chronic kidney disease; DN, diabetic nephropathy; eGFR, estimated glomerular filtration rate; ESA, erythropoiesis-stimulating agent; GN, glomerulonephritis; HTN, hypertension; OR, Odds ratio; PCR, protein to creatinine ratio; PKD, polycystic kidney disease.
(DOCX)
