# Supplementary material for: Albuminuria as a Risk Factor for Anemia in Chronic Kidney Disease: Result from the KoreaN Cohort Study for Outcomes in Patients With Chronic Kidney Disease (KNOW-CKD)
Source: PLoS One. 2015 Oct 2;10(10):e0139747. doi: 10.1371/journal.pone.0139747 (PMC4592200; doi:10.1371/journal.pone.0139747)
Supplement: S1 Table — Abbreviations: ACR, albumin to creatinine ratio; ACEI, angiotensin converting enzyme inhibitor; ARB, angiotensin receptor blocker; BMI, body mass index; CI, confidence interval; CKD, chronic kidney disease; DN, diabetic nephropathy; eGFR, estimated glomerular filtration rate; ESA, erythropoiesis-stimulating agent; GN, glomerulonephritis; HTN, hypertension; LVMI, left ventricular mass index; OR, Odds ratio; PCR, protein to creatinine ratio; PKD, polycystic kidney disease; PTH, parathyroid hormone; SBP, systolic blood pressure; TSAT, transferrin saturation. (DOCX) [file pone.0139747.s001.docx]

**S1 Table. Baseline demographics and the laboratory value according to the cause of CKD**

|  | GN | DN | HTN | PKD | P-value |
| --- | --- | --- | --- | --- | --- |
| Age | 49.6 ± 12.3 | 59.4 ± 9.3 | 59.7 ± 10.8 | 46.7 ± 10.4 | <0.001 |
| Female Sex | 226 (42.8%) | 104 (32.7%) | 82 (27.6%) | 25 (35.2%) | <0.001 |
| Smoking | 83 (15.7%) | 48 (15.1%) | 58 (19.5%) | 40 (15.6%) | 0.50 |
| ACR (mg/g) | 894 ± 1153 | 1762 ± 1918 | 493 ± 850 | 110 ± 198 | <0.01 |
| eGFR (mL/min per 1.73m^2^) | 59.3 ± 31.2 | 33.8 ± 19.2 | 38.3 ± 18.3 | 71.8 ± 33.5 | <0.01 |
| Anemia | 168 (32.5%) | 247 (78.2%) | 128 (43.2%) | 72 (28.2%) | <0.001 |
| TSAT |  |  |  |  | <0.01 |
| Quintile 1 (<21.5%) | 100 (19.1%) | 74 (23.6%) | 53 (18.1%) | 11 (15.7%) |  |
| Quintile 2 (21.5–27.0%) | 93 (17.8%) | 71 (22.6%) | 56 (19.1%) | 19 (27.1%) |  |
| Quintile 3 (27.1–32.7%) | 93 (17.8) | 67 (21.3%) | 65 (22.2%) | 10 (14.3%) |  |
| Quintile 4 (32.8–40.2%) | 103 (19.7%) | 64 (20.4%) | 57 (19.5%) | 16 (22.9%) |  |
| Quintile 5 (≥40.3%) | 134 (25.6%) | 38 (12.1%) | 41 (16.3%) | 14 (20.0%) |  |
| Ferritin |  |  |  |  | 0.02 |
| Quintile 1 (<45.8 ng/mL) | 117 (22.6%) | 51 (16.2%) | 50 (17.1%) | 7 (10.3%) |  |
| Quintile 2 (45.8–79.0 ng/mL) | 111 (21.4%) | 61 (19.4%) | 63 (21.6%) | 11 (16.2%) |  |
| Quintile 3 (79.1–121.6 ng/mL) | 103 (19.9%) | 65 (19.7%) | 61 (20.9%) | 16 (23.5%) |  |
| Quintile 4 (121.7–191.7 ng/mL) | 103 (19.9%) | 62 (19.7%) | 55 (18.8%) | 23 (33.8%) |  |
| Quintile 5 (>191.8 ng/mL) | 84 (16.2% | 76 (24.1%) | 63 (21.6%) | 11 (16.2%) |  |
| Albumin (g/dL) | 4.13 ± 0.39 | 3.99 ± 0.46 | 4.28 ± 0.31 | 4.40 ± 0.27 | <0.001 |
| Total cholesterol (mg/dL) | 179.3 ± 38.7 | 163.9 ± 39.7 | 169.1 ± 33.7 | 177.9 ± 32.1 | <0.001 |
| Calcium (mg/dL) | 9.13 ± 0.50 | 8.89 ± 0.63 | 9.17 ± 0.45 | 9.26 ± 0.45 | <0.001 |
| Phosphorus (mg/dL) | 3.61 ± 0.64 | 4.02 ± 0.73 | 3.60 ± 0.61 | 3.63 ± 0.59 | <0.001 |
| PTH (pg/mL) | 62.4 ± 62.7 | 100.8 ± 96.2 | 82.4 ± 72.0 | 61.4 ± 77.3 | <0.001 |
| LVMI (g/m^2^) | 88.8 ± 24.3 | 106.8 ± 30.9 | 101.0 ± 30.7 | 86.0 ± 20.2 | <0.001 |
| Use of ESA (versus non-users) | 33 (6.3%) | 54 (17.0%) | 24 (8.1%) | 13 (5.1%) | <0.001 |
| *Abbreviations*: ACR, albumin to creatinine ratio; ACEI, angiotensin converting enzyme inhibitor; ARB, angiotensin receptor blocker; BMI, body mass index; CI, confidence interval; CKD, chronic kidney disease; DN, diabetic nephropathy; eGFR, estimated glomerular filtration rate; ESA, erythropoiesis-stimulating agent; GN, glomerulonephritis; HTN, hypertension; LVMI, left ventricular mass index; OR, Odds ratio; PKD, polycystic kidney disease; PTH, parathyroid hormone; SBP, systolic blood pressure; TSAT, transferrin saturation. | | | | | |
