# Supplementary material for: Albuminuria as a Risk Factor for Anemia in Chronic Kidney Disease: Result from the KoreaN Cohort Study for Outcomes in Patients With Chronic Kidney Disease (KNOW-CKD)
Source: PLoS One. 2015 Oct 2;10(10):e0139747. doi: 10.1371/journal.pone.0139747 (PMC4592200; doi:10.1371/journal.pone.0139747)
Supplement: S2 Table — Abbreviations: ACR, albumin to creatinine ratio; ACEI, angiotensin converting enzyme inhibitor; ARB, angiotensin receptor blocker; BMI, body mass index; CI, confidence interval; CKD, chronic kidney disease; DN, diabetic nephropathy; eGFR, estimated glomerular filtration rate; ESA, erythropoiesis-stimulating agent; GN, glomerulonephritis; HTN, hypertension; LVMI, left ventricular mass index; OR, Odds ratio; PCR, protein to creatinine ratio; PKD, polycystic kidney disease; PTH, parathyroid hormone; SBP, systolic blood pressure; TSAT, transferrin saturation. (DOCX) [file pone.0139747.s002.docx]

**S2 Table. Univariate logistic regression analysis for the risk of anemia**

| Variables | Anemia | |
| --- | --- | --- |
|  | OR (95% CI) | P-value |
| Age (per 10 years) | 1.36 (1.25–1.49) | <0.001 |
| Female sex (versus male) | 1.55 (1.25–1.92) | <0.001 |
| Smokers (versus non-smokers) | 0.53 (0.40–0.81) | 0.001 |
| Cause of CKD |  |  |
| PKD | 1.00 (reference) |  |
| DN | 9.10 (6.21–13.33) | <0.001 |
| HTN | 1.93 (1.35–2.75) | <0.001 |
| GN | 1.22 (0.88–1.70) | 0.23 |
| BMI (kg/m^2^) | 0.95 (0.92–0.98) | <0.01 |
| SBP (per 5 mm Hg) | 1.06 (1.03–1.09) | <0.001 |
| Pulse pressure (per 5 mm Hg) | 1.16 (1.11–1.21) | <0.001 |
| eGFR categories |  |  |
| ≥60 | 1.00 (reference) |  |
| 45–59 | 2.55 (1.47–4.41) | <0.001 |
| 30–44 | 5.39 (3.16–9.20) | <0.001 |
| 15–29 | 9.01 (5.41–15.0) | <0.001 |
| <15 | 32.6 (19.2–55.2) | <0.001 |
| ACR categories |  |  |
| A1 (<30 mg/g) | 1.00 (reference) |  |
| A2 (30–299 mg/g) | 2.75 (1.88–4.01) | <0.001 |
| A3 (≥300 mg/g) | 4.91 (3.43–7.02) | <0.001 |
| Ferritin |  |  |
| Quintile 1 (<45.8 ng/mL) | 1.45 (1.04–2.02) | 0.03 |
| Quintile 2 (45.8–79.0 ng/mL) | 1.09 (0.78–1.52) | 0.62 |
| Quintile 3 (79.1–121.6 ng/mL) | 1.00 (reference) |  |
| Quintile 4 (121.7–191.7 ng/mL) | 1.10 (0.78–1.52) | 0.61 |
| Quintile 5 (>191.8 ng/mL) | 1.61 (1.16–2.44) | < 0.01 |
| TSAT |  |  |
| Quintile 1 (<21.5%) | 2.06 (1.48–2.88) | <0.001 |
| Quintile 2 (21.5–27.0%) | 1.14 (0.82–1.60) | 0.44 |
| Quintile 3 (27.1–32.7%) | 1.00 (reference) |  |
| Quintile 4 (32.8–40.2%) | 0.95 (0.68–1.32) | 0.74 |
| Quintile 5 (≥40.3%) | 0.61 (0.43–0.86) | < 0.01 |
| Albumin (g/dL) | 0.17 (0.12–0.23) | <0.001 |
| Total cholesterol (mg/dL) | 0.99 (0.99–1.00) | 0.001 |
| Calcium (mg/dL) | 0.22 (0.17–0.29) | <0.001 |
| Phosphorus (mg/dL) | 3.59 (2.94–4.35) | <0.001 |
| PTH (pg/mL) | 1.01 (1.01–1.02) | <0.001 |
| LVMI (g/m^2^) | 1.02 (1.01–1.02) | < 0.001 |
| Use of ESA (versus non-users) | 13.15 (7.33–23.59) | <0.001 |
| *Abbreviations*: ACR, albumin to creatinine ratio; ACEI, angiotensin converting enzyme inhibitor; ARB, angiotensin receptor blocker; BMI, body mass index; CI, confidence interval; CKD, chronic kidney disease; DN, diabetic nephropathy; eGFR, estimated glomerular filtration rate; ESA, erythropoiesis-stimulating agent; GN, glomerulonephritis; HTN, hypertension; LVMI, left ventricular mass index; OR, Odds ratio; PCR, protein to creatinine ratio; PKD, polycystic kidney disease; PTH, parathyroid hormone; SBP, systolic blood pressure; TSAT, transferrin saturation. | | |
