# Supplementary material for: Albuminuria as a Risk Factor for Anemia in Chronic Kidney Disease: Result from the KoreaN Cohort Study for Outcomes in Patients With Chronic Kidney Disease (KNOW-CKD)
Source: PLoS One. 2015 Oct 2;10(10):e0139747. doi: 10.1371/journal.pone.0139747 (PMC4592200; doi:10.1371/journal.pone.0139747)
Supplement: S3 Table — Anemia (hemoglobin <13 g/dL for men, <12 g/dL for women or with use of ESA). Model 1: adjusted for age and sex. Model 2: adjusted for age, sex, and the eGFR. Model 3: adjusted for age, sex, the eGFR, serum calcium level, BMI, use of an ESA, smoking, the cause of CKD, and ferritin level. Abbreviations: ACR, albumin creatinine ratio; eGFR, estimated GFR; CI, confidence interval; BMI, body mass index; ESA, erythropoiesis stimulating agent; CKD, chronic kidney disease. (DOCX) [file pone.0139747.s003.docx]

**S3 Table. Odds ratio for anemia associated with ACR**

|  | Prevalence rates | OR (95% CI) | | |  |
| --- | --- | --- | --- | --- | --- |
| ACR (mg/g) | N (%) | Model 1 | Model 2 | Model 3 | |
| <30 | 45 (19.8%) | 1 (reference) | 1 (reference) | 1 (reference) |  |
| 30–299 | 186 (40.9%) | 2.74 (1.87–4.02) | 1.47 (0.96–2.26) | 1.44 (0.87–2.40) |  |
| ≥300 | 426 (55.0%) | 5.06 (3.52–7.28) | 2.11 (1.40–3.17) | 1.84 (1.08–3.14) |  |
| Anemia (hemoglobin <13 g/dL for men, <12 g/dL for women or with use of ESA)  Model 1: adjusted for age and sex  Model 2: adjusted for age, sex, and the eGFR  Model 3: adjusted for age, sex, the eGFR, serum calcium level, BMI, use of an ESA, smoking, the cause of CKD, and ferritin level  *Abbreviations*: ACR, albumin creatinine ratio; eGFR, estimated GFR; CI, confidence interval; BMI, body mass index; ESA, erythropoiesis stimulating agent; CKD, chronic kidney disease. | | | | |  |
