# Supplementary material for: Albuminuria as a Risk Factor for Anemia in Chronic Kidney Disease: Result from the KoreaN Cohort Study for Outcomes in Patients With Chronic Kidney Disease (KNOW-CKD)
Source: PLoS One. 2015 Oct 2;10(10):e0139747. doi: 10.1371/journal.pone.0139747 (PMC4592200; doi:10.1371/journal.pone.0139747)
Supplement: S4 Table — Anemia (hemoglobin <13 g/dL for men, <12 g/dL for women). Model 1: adjusted for age and sex. Model 2: adjusted for age, sex, and the eGFR.Model 3: adjusted for age, sex, the eGFR, serum calcium level, BMI, use of an ESA, smoking, the cause of CKD, and ferritin level. Abbreviations: ACR, albumin creatinine ratio; eGFR, estimated GFR; CI, confidence interval; BMI, body mass index; ESA, erythropoiesis stimulating agent; CKD, chronic kidney disease. (DOCX) [file pone.0139747.s004.docx]

**S4 Table. Odds ratio for anemia associated with ACR (patients with ESA are excluded)**

|  | Prevalence rates | OR (95% CI) | | |  |
| --- | --- | --- | --- | --- | --- |
| ACR (mg/g) | N (%) | Model 1 | Model 2 | Model 3 | |
| <30 | 38 (17.4%) | 1 (reference) | 1 (reference) | 1 (reference) |  |
| 30–299 | 157 (37.0%) | 2.78 (1.85–4.18) | 1.58 (1.01–2.47) | 1.44 (0.86–2.40) |  |
| ≥300 | 335 (49.1%) | 4.73 (3.21–6.96) | 2.25 (1.47–3.44) | 1.84 (1.08–3.14) |  |
| Anemia (hemoglobin <13 g/dL for men, <12 g/dL for women).  Model 1: adjusted for age and sex  Model 2: adjusted for age, sex, and the eGFR  Model 3: adjusted for age, sex, the eGFR, serum calcium level, BMI, use of an ESA, smoking, the cause of CKD, and ferritin level  *Abbreviations*: ACR, albumin creatinine ratio; eGFR, estimated GFR; CI, confidence interval; BMI, body mass index; ESA, erythropoiesis stimulating agent; CKD, chronic kidney disease. | | | | |  |
