# Supplementary material for: Albuminuria as a Risk Factor for Anemia in Chronic Kidney Disease: Result from the KoreaN Cohort Study for Outcomes in Patients With Chronic Kidney Disease (KNOW-CKD)
Source: PLoS One. 2015 Oct 2;10(10):e0139747. doi: 10.1371/journal.pone.0139747 (PMC4592200; doi:10.1371/journal.pone.0139747)
Supplement: S5 Table — Abbreviations: ACR, albumin to creatinine ratio; BMI, body mass index; CI, confidence interval; CKD, chronic kidney disease; DN, diabetic nephropathy; eGFR, estimated glomerular filtration rate; ESA, erythropoiesis-stimulating agent; GN, glomerulonephritis; HTN, hypertension; OR, Odds ratio; PCR, protein to creatinine ratio; PKD, polycystic kidney disease. (DOCX) [file pone.0139747.s005.docx]

**S5 Table. Multivariate logistic regression analysis for the risk of anemia**

| Variables | Anemia | |
| --- | --- | --- |
|  | OR (95% CI) | P-value |
| Age (per 10 years) | 1.05 (0.91–1.20) | 0.51 |
| Female sex (versus male) | 1.59 (1.15–2.21) | <0.001 |
| Smoking (versus non-smokers) | 0.52 (0.34–0.80) | <0.01 |
| Cause of CKD |  |  |
| PKD | 1.00 (reference) |  |
| DN | 3.67 (2.13–6.32) | <0.001 |
| HTN | 0.87 (0.52–1.43) | 0.57 |
| GN | 0.62 (0.39–0.99) | 0.05 |
| eGFR (mL/min per 1.73m^2^) |  |  |
| ≥60 | 1.00 (reference) |  |
| 45–59 | 2.81 (1.75–4.51) | <0.001 |
| 30–44 | 4.21 (2.67–6.64) | <0.001 |
| 15–29 | 15.2 (9.44–24.4) | <0.001 |
| <15 | 31.7 (13.3–75.6) | <0.001 |
| ACR (mg/g) |  |  |
| <30 | 1.00 (reference) |  |
| 30–299 | 1.43 (0.88–2.33) | <0.001 |
| ≥300 | 1.86 (1.12–3.10) | <0.001 |
| BMI (kg/m^2^) | 0.91 (0.87–0.95) | <0.01 |
| Serum ferritin |  |  |
| Quintile 1 (<45.8 ng/mL) | 1.84 (1.17–2.87) | <0.01 |
| Quintile 2 (45.8–79.0 ng/mL) | 1.04 (0.67–1.62) | 0.87 |
| Quintile 3 (79.1–121.6 ng/mL) | 1.00 (reference) |  |
| Quintile 4 (121.7–191.7 ng/mL) | 1.14 (0.71–2.65) | 0.59 |
| Quintile 5 (>191.8 ng/mL) | 1.66 (1.04–2.65) | <0.05 |
| Use of ESA (versus non-users) | 2.00 (1.00–3.99) | 0.05 |
| Serum calcium (mg/dL) | 0.38 (0.28–0.53) | <0.001 |
| *Abbreviations*: ACR, albumin to creatinine ratio; BMI, body mass index; CI, confidence interval; CKD, chronic kidney disease; DN, diabetic nephropathy; eGFR, estimated glomerular filtration rate; ESA, erythropoiesis-stimulating agent; GN, glomerulonephritis; HTN, hypertension; OR, Odds ratio; PCR, protein to creatinine ratio; PKD, polycystic kidney disease. | | |
